# Cerebral Haemodynamic Assessment Following Sport-related Concussion (Mild Traumatic Brain Injury) in Youth and Amateur Rugby Union Players

**DOI:** 10.1186/s40798-025-00849-2

**Published:** 2025-05-02

**Authors:** Ben Jones, Mohammadreza Jamalifard, Sally Waterworth, Mike Rogerson, Javier Andreu-Perez, Jay Perrett, Edward Hope, Jason Moran, Tom Adams, Jyotpal Singh, Patrick Neary, Chris E. Cooper

**Affiliations:** 1 School of Sport Rehabilitation and Exercise Sciences, University of Essex, Colchester, UK; 2 School of Computer Science and Electronic Engineering, University of Essex, Colchester, UK; 3 PhysiGo, Carlton Business Centre, Wiltshire, UK; 4 School of Sport and Exercise Sciences, Liverpool John Moores University, Liverpool, UK; 5 Faculty of Kinesiology & Health Studies, University of Regina, Regina, SK S4S 1A2 Canada

**Keywords:** Functional Near-Infrared Spectroscopy, Concussion, Rugby, Graduated Return To Play

## Abstract

**Background:**

Using functional near-infrared spectroscopy (fNIRS) as an objective diagnostic tool, we aimed to (1) compare fNIRS measurements in adult and youth male rugby players against controls over a playing season, and 2) document the fNIRS changes that occur in concussed rugby players during the England Rugby Football Union Graduated Return-To-Play programme (GRTP). Sixty-seven participants (rugby = 41 (26 adults: 27.5 ± 4.4 years; 15 youth: 16.5 ± 0.6 years; control = 26 (11 adult: 30.5 ± 5.2 years; 15 youth: 16.9 ± 0.4 years) completed fNIRS assessments at pre, mid and end-season. Eight players (five youth, three adult) sustained concussions, and completed fNIRS and the Graded Symptom Checklist from the Sport Concussion Assessment Tool version 5 (SCAT5) assessment throughout the GRTP period. Mixed linear models were utilised to assess the effect of group and time on fNIRS measures of oxyhaemoglobin (∆O_2_Hb) and deoxyhaemoglobin (∆HHb) during performance tasks. Typical Error (TE) i.e., normal biological fluctuation and measurement error, was calculated to identify ‘cut-off’ thresholds for identifying effects of concussion.

**Results:**

There were significant differences in fNIRS indices over time in adult and youth groups (*p* < 0.05) but no significant differences between rugby and control groups (*p* > 0.05). Seven out of eight (87.5%) concussed players showed changes greater than TE during the GRTP period for both ∆O_2_Hb and ∆HHb during performance tasks and these players’ ∆O_2_Hb profiles had not returned to within ‘normal’ levels within the GRTP period. All players’ symptom severity and number returned to normal within the GRTP period.

**Conclusion:**

Current GRTP protocols alone are problematic and there is a need for a more individualised approach to concussion management, utilising objective biomarker tools such as fNIRS.

**Supplementary Information:**

The online version contains supplementary material available at 10.1186/s40798-025-00849-2.

## Key Points

• Graduated return-to-play protocols are primarily based on subjective, symptom-based reporting despite limitations.

• Functional Near-Infrared Spectroscopy (fNIRS) can track recovery time course following concussion.

• fNIRS responses remained altered, even after resolution of subjective symptoms.

• Traditional clinical clearance methods might not fully capture the brain’s neurophysiological recovery, potentially exposing players to increased risk of subsequent injuries.

• Incorporating objective measures such as fNIRS into return-to-play protocols could enhance the safety and health outcomes of rugby players.

**Supplementary Information:**

The online version contains supplementary material available at 10.1186/s40798-025-00849-2.

## Background

Sport-related concussion (SRC) is a mild traumatic brain injury (mTBI) caused by external forces applied to the brain [1]. Repetitive concussive injuries and sub-concussive impacts can increase the risk of neurodegenerative complications ranging from mild brain impairments to neurological disease such as dementia and chronic traumatic encephalopathy (CTE) [2]. In certain sports (e.g. rugby, American Football, boxing), risk of concussion is inherent [3, 4]. Over the course of one season, Rugby Union players may experience up to 100 direct head impacts [5] and reporting of concussion incidence in professional rugby has risen significantly from 2009 to 2019 [6]. This trend may not only reflect an increase in the physicality of contact or combat sports but could also be attributed to greater concussion awareness and improved head injury assessment reporting. As such research to improve the understanding of risks, methods for diagnosis and, most importantly, improving the long-term brain consequences for those affected is needed [7, 8].

Near-Infrared Spectroscopy (NIRS) provides measurements of relative changes in blood oxygenation [9]. Functional near-infrared spectroscopy (fNIRS) has been shown to be a valid technique for measuring cerebrovascular health [8] and has demonstrated utility in monitoring concussion recovery [9–11]. For example, retired contact sport players with a history of concussion exhibit altered cortical activity and impaired dynamic cerebral autoregulation (dCA) compared to age-matched controls [12–14]. Research has reported elevated oxygenated haemoglobin (O_2_Hb) responses in the prefrontal cortex (PFC) during repeated squat-stand manoeuvres, indicative of compensatory mechanisms for impaired perfusion pressure regulation [12]. Additionally, studies have observed reduced total haemoglobin (tHb), a surrogate marker for cerebral blood volume during visual search tasks that induce Neurovascular Coupling (NVC), reflecting impaired oxygen delivery [13]. Retired rugby players exhibit reduced O_2_Hb and increased deoxygenated haemoglobin (HHb) during cognitive tasks, suggesting long-term impacts on NVC [14]. Sub-acute and acute impacts across various combat [15, 16] and contact sports [17, 18] have also shown altered cerebral responses. Adolescent soccer players demonstrated increased O_2_Hb in the PFC during complex balance and cognitive tasks following a season of repetitive head impact exposure, suggesting compensatory prefrontal activation to maintain performance [18]. Similarly, university athletes showed acutely increased O_2_Hb responses following ball heading during single and dual-task assessments, indicative of heightened prefrontal recruitment for motor-cognitive responses [17]. In professional boxers, significant cortical deoxygenation during orthostatic challenges highlighted impaired cerebral oxygenation and dCA due to repetitive trauma [16]. Recently, Martini et al. [10] and Jain et al. [11], have demonstrated the sensitivity of fNIRS to detect persistent alterations in prefrontal cortical activity during gait and dual-task challenges in concussed adolescents compared to controls. Consequentially, these findings support the use of fNIRS as an objective measurement tool for concussion [12]. Additionally, fNIRS derived measurements of NVC and dCA may function as biomarkers of cerebrovascular health, aiding in concussion diagnosis and return-to-play/learn processes.

The purpose(s) of this study were to; (1) compare fNIRS measurements following NVC and dCA inducing tasks in adult and youth male rugby players and non-contact sport controls over a playing season and; (2) document the fNIRS changes that occur in concussed rugby players during the 2022-23 England Rugby Football Union (RFU) Graduated Return to-To-Play (GRTP) programme [13].

We hypothesised that; (1) fNIRS changes in male rugby players over a season would be significantly different from those in a control group; (2) rugby players who sustain a concussion will exhibit more pronounced fNIRS changes than non-concussed players.

## Methods

A longitudinal study was used to characterise the effects of a season of rugby union on cerebral oxygenation. fNIRS data were collected at: pre, mid, end-season. An exploratory, prospective cohort study utilising rugby players who sustained a concussion was used to document the fNIRS changes that occur during the England RFU GRTP programme [13].

### Participants

Participants were adult male 1st XV and youth players (16–18 years) from a UK amateur rugby club and controls. Control group participants were recruited from local sports clubs and schools, did not take part in contact or combat sports and had no history of concussion in the last 5 years. Contact or combat sports were defined as sports or activities where participants engaged in physical interactions that involve grappling, striking or forceful body contact [14]. All participants completed a General Health Questionnaire (GHQ). Demographic information is shown in Table 1.

**Table 1:** Group demographic and general health questionnaire data (mean ± SD)

Variable	Adult Rugby	Adult Control	Youth Rugby	Youth Control
Participants (*n*)	26	11	15	15
Age (years)	27.5 ± 4.4	30.5 ± 5.2	16.5 ± 0.6	16.9 ± 0.4
Height (cm)	181.4 ± 5.8	180.2 ± 6.9	181.1 ± 5.0*	174.2 ± 5.7
Body Mass (kg)	103.0 ± 17.0*	82.1 ± 9.1	83.7 ± 18.6*	69.8 ± 14.2
Previous Concussions	2.3 ± 2.1*	0	1.9 ± 2.8*	0
Physical Activity (mins/week)	514.2 ± 286.9	533.8 ± 349.8	588.0 ± 271.98*	295.3 ± 136.9
Games played	12.5 ± 5.2	N/A	12.1 ± 7.5	N/A
Minutes Played (mins)	866.2 ± 375.5*	N/A	621.8 ± 337.8	N/A

Note: Control subjects’ various sports included baseball, 5-aside football (no heading), powerlifting, swimming, and running

*Significant differences between rugby and control groups

### Procedures

Testing took place in the same set of rooms at the same time of day. Participants had not exercised in the previous 12-hours or consumed alcohol/ caffeine within 6-hours. Baseline testing was conducted over two days. Day one involved demographic measures, GHQ, and the Sport Concussion Assessment Tool v5 (SCAT5) (adult rugby only). On day two, fNIRS assessment took place.

Any concussions were identified on field by team medical (physiotherapist) or coaching staff. Following the 2022-23 England RFU GRTP programme guidelines [13], clinical recovery was defined as a resolution of post-concussion symptoms, and resumption of normal activities (school/work/sport).

England RFU GRTP guidelines during the 2022/23 playing season followed the same six stage return to play process as the World Rugby GRTP programme [17], with longer mandatory stand down periods of initial rest i.e., youth players (U19) minimum of 23 days and adult community players 21 days.

The SCAT5 is a standardised tool for evaluating concussions [1, 15]. It was administered to adult rugby players during pre-season assessments. If a concussion occurred, symptom evaluation (number and severity) from the Graded Symptom Checklist (GSC) was repeated during the GRTP process. The SCAT5 has been shown to discriminate between non-concussed and concussed athletes [1, 15], and is most valid within 72 h of concussion [1].

### fNIRS Device and Protocol

**Fig. 1:**
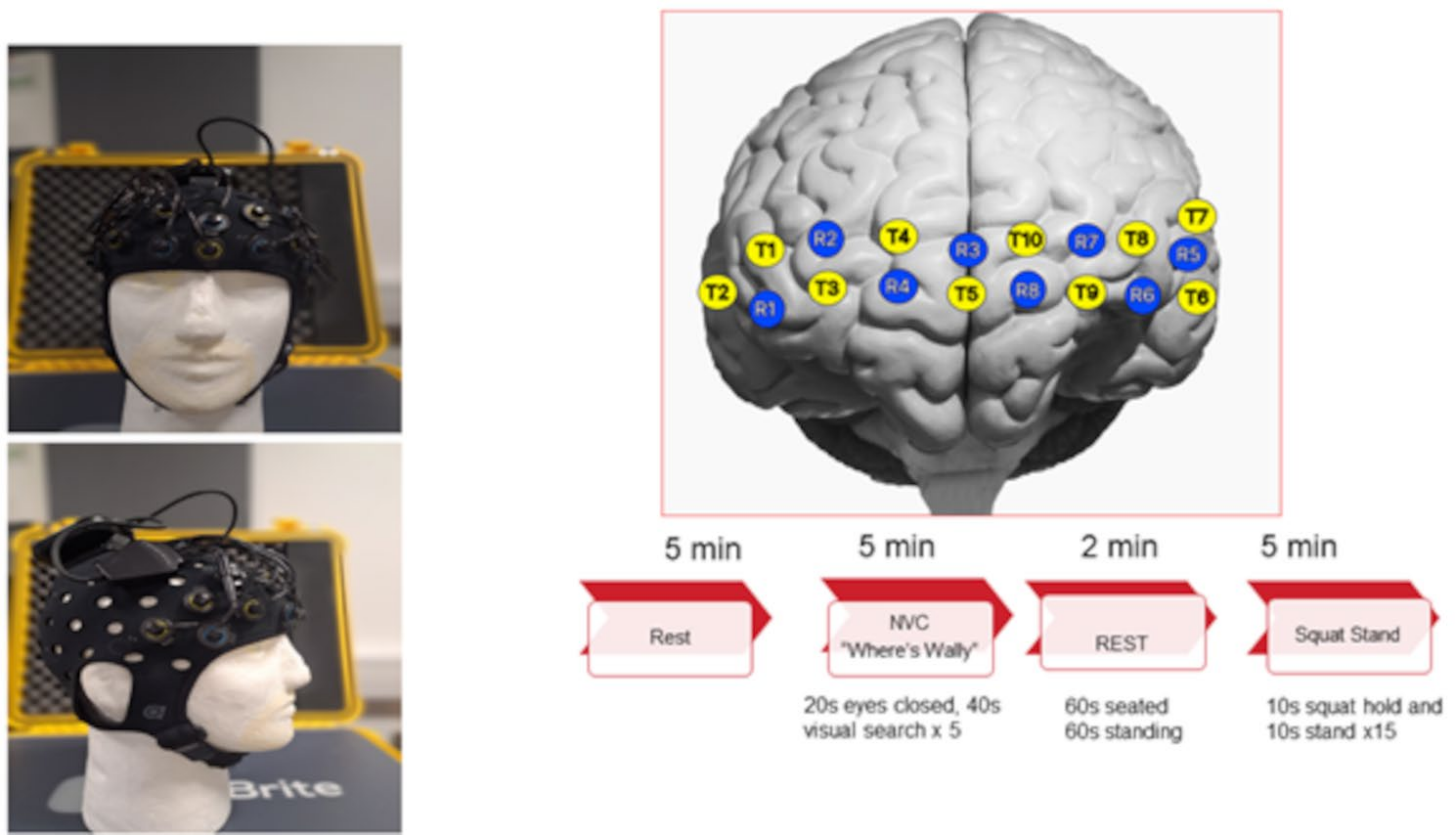
Headcap frontal and lateral views and 3D plot depicting optode arrangement and experimental protocol. T = transmitter and R = receiver. Data were collected at a sample frequency of 50 Hz via multi-wavelength LEDs situated on a soft neoprene head-cap containing 8 receivers and 10 transmitter channel pairs, 30 mm apart. The probe positions of the fNIRS detection device covered the area linking Fp1, F3, F7, and Fp2, F4, F8, corresponding to the left and right prefrontal cortex respectively, according to the international EEG 10–20 system [21]

The fNIRS head cap was placed 1 cm above the eyebrows on the supraorbital ridge to avoid the sinuses (Fig. 1). The 24-channel, continuous wave system (Brite, Artinis Medical Systems, Elst, The Netherlands, www.artinis.com) monitored changes (micro-molar, µM) in oxyhaemoglobin (∆O_2_Hb), and deoxyhaemoglobin (∆HHb) in the pre-frontal cortex (PFC). Changes in light attenuation were measured at two wavelengths (762 and 843 nm) and concentration change was calculated using the modified Beer–Lambert law within Oxysoft software (v3.5.15.2.). The differential path length factor (DPF) was calculated for individuals in relation to their age [16].

A modified Neary Protocol, detailed elsewhere [7], was used to assess brain oxygenation during tasks that induced NVC using the “Where’s Wally” visual search task paradigm and dynamic cerebral autoregulation (dCA) was assessed using a squat-stand (SS) manoeuvre. Briefly, the ‘‘Where’s Wally’’ test was conducted on a high definition (HD) screen (22"size), with participants at a self-selected distance for optimal focus. A block-design was utilised, consisting of five cycles of 20 s with eyes closed followed by 40 s with eyes open during which the participant searched the screen for “Wally”. If the participant found ‘‘Wally” the slide was immediately advanced to a new slide. This continued for the duration of each 40 s eyes open block. The test took 5 minutes in total. Prior to the SS manoeuvre, the participant sat quietly for 1 minute and then stood up for 1 minute to allow the body to adjust to a standing position. Participants then completed 5 minutes of a SS manoeuvre which consisted of 10 s squatting, followed by 10 s standing (total of 15 squats) [18]. During the squat portion, participants held a ∼ 90° knee angle, and then returned to standing at a pace dictated by an audio timer on the HD screen. Previous research have shown these tests to be valid measures of NVC [19] and dCA [20] respectively. Task Performance was evaluated by the number of correct object identifications during the “Where’s Wally” task.

### fNIRS Data Analysis

fNIRS signals were exported into MNE-NIRS, a Python toolbox, for analysis [22]. Each channel was visually inspected for signal quality. Channels with excessive motion artifacts, and/or non-pulsatile signals were excluded [23] utilising the signal quality index (SQI) function (i.e. SQI Value < 3) implemented in Python [24]. A 5th order Butterworth filter was utilised to remove motion artifact [25, 26] and a low pass filter (cut-off frequency of 0.09 Hz) was applied to all channels to keep all frequencies below the lowest systemic frequency [27]. Across all participants and groups, an average of 3% of channels were discarded at the pre-season (range of 0–8%), 4% at mid-season (range of 0–13%), and 4% at end-season (range 0–17%).

To evaluate changes in PFC oxygenation, data across all 24 channels covering the PFC were averaged. This approach was chosen to capture global cortical trends, reflecting the overall activation of the PFC during tasks that induce NVC and dCA.

O_2_Hb and HHb variables were analysed by calculating the change (Δ) between the average maximal values during the 40-second eyes-open task and the average minimal values during the 20-second eyes-closed within each trial for the “Where’s Wally” visual search task. This computation was repeated across five trials for each channel, and the resulting Δ values were averaged across the 24 channels. For the squat-stand manoeuvre, Δ values were similarly calculated as the difference between the average maximal values during the 10-second squat and the average minimal values during the 10-second stand periods, averaged across the 15 trials and 24 channels [28, 29].

### Statistical Analysis

Descriptive statistics are presented as mean ± SD (Table 1). Descriptive comparisons were analysed using independent t-tests. Separate mixed-effects linear models were used to assess the effect of group (control, rugby) and time point (pre-season, mid-season, and end-season) on ∆O_2_Hb and ∆HHb during the “Where’s Wally” and squat-stand manoeuvre tasks in adult and youth rugby and control cohorts. Models were fitted using Restricted Maximum Likelihood estimation. If statistically significant differences were identified, Bonferroni post hoc tests were applied to correct for multiple comparisons. Given the known protracted development of the prefrontal cortex, which continues to mature into the mid-20s [30, 31] and recent evidence that has indicated concussed adolescents show different responses in NVC compared to adults [32] youth (16.6 ± 0.5 years) and adult (28.4 ± 4.8 years) cohorts were analysed separately to account for age-related differences in neural activation and cognitive performance during working memory tasks. We used repeated measures taken across the playing season from rugby and control participants to calculate Typical Error (TE) represents the variability in observed measurements due to both instrumentation noise (e.g., equipment precision) and biological noise (e.g., circadian influences, hydration status) [33]. It was calculated as the standard deviation of the differences between repeated measurements divided by √2​.

Equation:

$$\frac{{TE = SDdiff}}{{\sqrt 2 }}$$

In this study, repeated measures from only non-concussed rugby and control participants were analysed using the Hopkins reliability spreadsheet to calculate TE [32]. Data are reported as means with 90% confidence intervals (CI). TE values for fNIRS ΔO_2_Hb, ΔHHb, “Where’s Wally,” and squat-stand were calculated separately for each group (adult and youth, rugby and control participants). These TE thresholds were subsequently used to distinguish meaningful changes in concussed rugby players, beyond the range of normal biological and measurement variability. Independent t-tests were employed to identify differences in TE values between rugby and control groups, establishing group-specific thresholds for further analysis [34]. These values served as thresholds for identifying effects of concussions beyond normal fluctuation / measurement error in concussed rugby players. Statistical analyses were performed using IBM SPSS v.29.0.

## Results

**Fig. 2:**
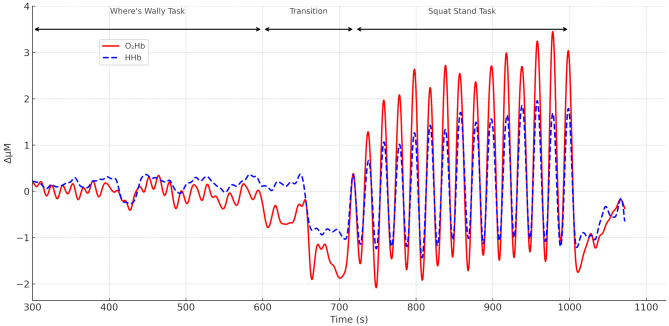
An individual oxyhaemoglobin (O_2_Hb) (solid line) and deoxyhaemoglobin (HHb) (dashed line) signal trace for an adult rugby player at pre-season. The signal profile is similar for all groups and time points within the season

Significant differences in baseline characteristics between rugby and control groups were identified by independent sample t-tests with significant differences in height (youth rugby taller than youth controls *p* = 0.001), and body mass (youth and adult rugby players heavier than controls *p* = 0.02, *p* = 0.001 respectively). Significant differences were also seen in concussion history with adult and youth rugby players having experienced a greater number of concussions compared to controls (*p* = 0.0009, *p* = 0.0042 respectively. Adult rugby players played significantly more minutes than youth players (*p* = 0.04: Table 1) Fig. 2 displays an example participant fNIRS timeline trace for O_2_Hb and HHb responses during the WW, transition and squat-stand tasks.

### Playing Season

Changes in fNIRS responses for control and rugby adult and youth groups during neurocognitive visual search (“Where’s Wally”) and neurophysiological (squat-stand) tasks are shown in Table 2.

**Table 2:** Adult and youth, control and rugby group mean ± se change (∆) in “where’s Wally” (WW) and squat-stand (SS) tasks; Oxyhaemoglobin (∆O_2_Hb) and Deoxyhaemoglobin (∆HHb) profiles across a rugby playing season at three time points (pre, mid and end season)

Age Group	Group	Task	Pre-Season		Mid-Season		End-Season	
			ΔO_2_Hb (µM) Mean ± SD	ΔHHb (µM) Mean ± SD	ΔO_2_Hb (µM) Mean ± SD	ΔHHb (µM) Mean ± SD	ΔO_2_Hb (µM) Mean ± SD	ΔHHb (µM) Mean ± SD
Adult	Control	WW	1.06 ± 0.55	0.25 ± 0.09	0.92 ± 0.50	0.25 ± 0.08	0.88 ± 0.45	0.27 ± 0.10
Adult	Rugby	WW	1.06 ± 0.63	0.28 ± 0.10	1.07 ± 0.44	0.28 ± 0.10	1.43 ± 0.84	0.38 ± 0.16
Youth	Control	WW	1.17 ± 0.73	0.32 ± 0.17	1.24 ± 0.76	0.31 ± 0.13*	1.21 ± 0.40	0.26 ± 0.10
Youth	Rugby	WW	0.89 ± 0.61	0.24 ± 0.11	1.14 ± 0.63	0.33 ± 0.12*	0.88 ± 0.50	0.23 ± 0.08
Adult	Control	SS	6.75 ± 1.81	1.05 ± 0.38	6.55 ± 1.62	0.98 ± 0.39	6.66 ± 2.46	1.07 ± 0.52
Adult	Rugby	SS	7.28 ± 1.97	1.17 ± 0.32	7.81 ± 1.88	1.28 ± 0.44	7.29 ± 2.36	1.26 ± 0.43
Youth	Control	SS	5.79 ± 2.83*	0.99 ± 0.50*	7.79 ± 3.31	1.28 ± 0.46†	7.00 ± 3.09	1.21 ± 0.44
Youth	Rugby	SS	6.10 ± 3.07*	1.08 ± 0.53*	7.57 ± 2.79	1.26 ± 0.53	8.21 ± 2.64	1.40 ± 0.41

*Significant change over time. †significant group x time interaction effect

### Neurocognitive: “Where’s Wally”

There was no significant effect of group (adult / youth control vs. rugby), time or interaction effect on ΔO_2_Hb (*p* > 0.05). There was no significant effect of group (adult / youth control vs. rugby), time (adult) or interaction effect on ∆HHb (*p* > 0.05) There was a significant effect of time on ∆HHb for youth groups (*p* = 0.04). In youth groups post-hoc pairwise comparisons indicated that mid-season ∆HHb was significantly higher compared to end-season.

### Task Performance: “Where’s Wally” Identification

There was no significant difference in “Where’s Wally” object identification between pre, mid and end season for all groups (*p* = > 0.05) (Supplemental 1).

### Neurophysiological: Squat-Stand Task

There was no significant effect of group (adult control vs. rugby), time or interaction effect on ∆O_2_Hb or ΔHHb (*p* > 0.05). There was no significant effect of group (youth control vs. rugby), or interaction effect on ∆O_2_Hb (*p* > 0.05). Time had a significant effect on ∆O_2_Hb (*p* = 0.004) and time (*p* = 0.03) and group by time interaction effect on ΔHHb (*p* = 0.02). Post-hoc pairwise comparisons indicated pre-season ∆O_2_Hb was significantly lower compared to mid-season (*p* = 0.013) and end-season (*p* = 0.01). ΔHHb pre-season was significantly lower than end-season (*p* = 0.03). The significant interaction effect showed that ΔHHb for the youth control group was higher at mid-season compared to youth rugby (*p* = 0.02).

### Typical Error (TE)

Calculated TE thresholds for ∆O_2_Hb and ∆HHb during “Where’s Wally” and squat-stand tasks are shown in Table 3. Independent samples t-tests showed significant differences between control and rugby participants TE scores for ∆HHb (adult) (*p* = 0.03) and ∆O_2_Hb (youth) (*p* = 0.001) “Where’s Wally” and no significant differences (*p* > 0.05) for SS (∆O_2_Hb and ∆HHb) respectively. Therefore, rugby participant only TE values were created for the adult ∆HHb and youth ∆O_2_Hb WW task and non-significant TE values were pooled between control and rugby participants for the ∆O_2_Hb adult and youth ∆HHb for WW and adult and youth squat-stand ∆O_2_Hb and ∆HHb to establish ‘cut-off’ TE threshold values (Table 3).

**Table 3:** Calculated typical error (TE) values for “where’s Wally” (WW) and squat stand (SS). Data are shown as mean with 90% confidence intervals (90%CI)

Group	O_2_Hb TE	HHb TE
Adult Rugby (WW)	0.29 [0.24, 0.37] +	0.10 [0.08, 0.13]
Youth Rugby (WW)	0.42 [0.30, 0.95]	0.11 [0.09, 0.14] +
Combined Adult (SS)	1.54 [1.31, 1.90] +	0.27 [0.23, 0.34] +
Combined Youth (SS)	1.41 [1.19, 1.80] +	0.23 [0.19, 0.29] +

All TE values can be found in Supplemental 2. + indicates pooled TE values

### Concussion

**Fig. 3:**
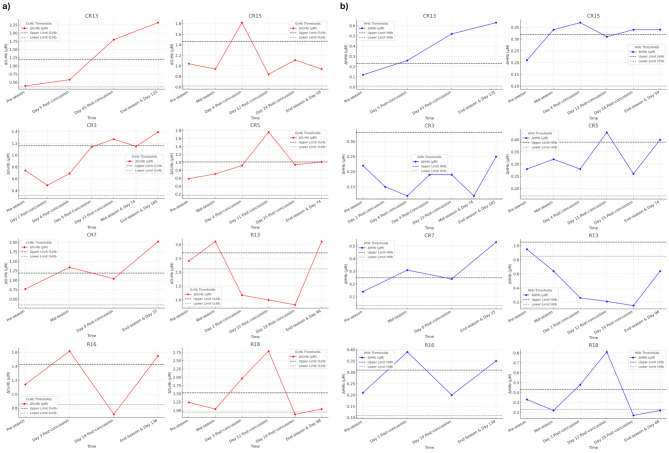
(**a**) Pre-season and post-concussion change in oxyhaemoglobin (∆O_2_Hb) for eight players (five youth, three adult). Seven out of eight (87.5%) players show changes greater than Typical Error (TE) during the early post-injury period (< 25 days) period. Six players show an increased ∆O_2_Hb response during the “Where’s Wally” task and one player shows a decrease. Findings indicate a dysregulated ∆O_2_Hb response following concussion. Six out of eight (75%) players’ ∆O_2_Hb profiles had not returned to within ‘normal’ levels within the GRTP period. R = adult rugby and CR = youth rugby. (**b**) Pre-season and post-concussion change in deoxyhaemoglobin (∆HHb) for eight players (five youth, three adult). Seven out of eight (87.5%) players show changes greater than TE during the early post-injury period (< 25 days) period. Six players show an increased ∆HHb response during the “Where’s Wally” task and one player shows a decrease. Findings indicate dysregulated ∆HHb responses following concussion. Five out of seven (71%) players’ ∆HHb profiles had not returned to within ‘normal’ levels within the GRTP period. R = adult rugby and CR = youth rugby

**Fig. 4:**
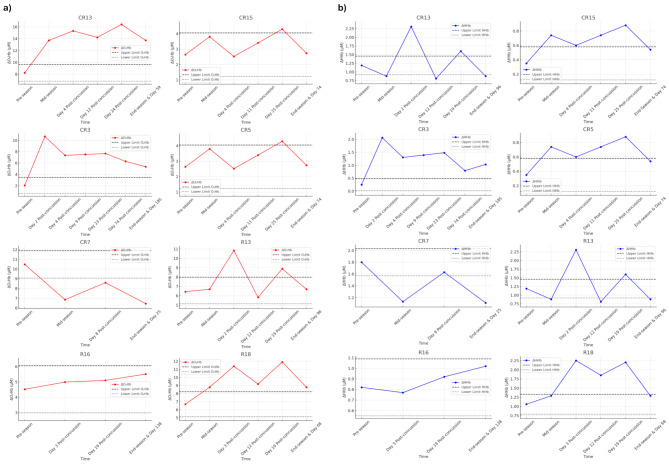
(**a**) Pre-season and post-concussion change in oxyhaemoglobin (∆O_2_Hb) for eight players (five youth, three adult). Seven (87.5%) players show changes greater than Typical Error (TE) during the early post-injury (< 25 days) period. Six players show increased ∆O_2_Hb responses during the squat stand manoeuvre and one player shows a decrease. Findings indicate a dysregulated ∆O_2_Hb response following concussion. Seven out of eight (87.5%) players’ ∆O_2_Hb profiles had not returned to within ‘normal’ levels within the GRTP period. R = adult rugby and CR = youth rugby. (**b**) Pre-season and post-concussion change in deoxyhaemoglobin (∆HHb) for eight players (five youth, three adult). Seven (87.5%) players show changes greater than TE during the early post-injury (< 25 days) period. Six players show increased ∆HHb responses during the squat stand manoeuvre and one player shows a decrease. Findings indicate dysregulated ∆HHb responses following concussion. Seven out of eight (87.5%) players’ ∆HHb profiles had not returned to within ‘normal’ levels within the GRTP period. R = adult rugby and CR = youth rugby

Individual concussion fNIRS profiles are shown for “Where’s Wally” (Fig. 3a and b) and squat-stand (Fig. 4a and b) tasks, with TE thresholds overlaid.

Concussion symptom number and severity reporting are shown in Table 4.

**Table 4:** Shows the graded symptom checklist (GSC) from sport concussion assessment tool version 5 (SCAT5) symptom severity and symptom number scores during the early post-injury (< 25 days) period

Participant ID	Visit 1 (*n* = 8)		Visit 2 (*n* = 8)		Visit 3 (*n* = 5)		Visit 4 (*n* = 1)	
	Symptom No.	Symptom Severity	Symptom No.	Symptom Severity	Symptom No.	Symptom Severity	Symptom No.	Symptom Severity
CR13	15*	39*	2	3				
CR15	21*	56*	10*	13	5	5		
CR3	14*	33*	7	10	3	3	1	1
CR5	9	17*	12*	23*	8	9		
CR7	12*	16*	4	4				
R13	15*	35*	0	0	0	0		
R16	0	0	0	0				
R18	4	6	1	1	0	0		
No. of Days	4 ± 2		1 7 ± 12		19 ± 6		23 ± 0	

Note: n = indicates the number of participants at each visit. Days indicates the mean numbers of days since concussion ± standard deviation

Symptom severity scale = 0-132; 0–14 ‘broadly normal’, 15–22 ‘above or below average’, 23–39 ‘unusually low or high’, 40–132 ‘extremely low or high’. Symptom number scale = 0–22; 0–19 ‘broadly normal’, 10–13 ‘above or below average’, 14–18 ‘unusually low or high’, 19–22 ‘extremely low or high’

* Indicates results greater than broadly normal [35]

Eight players (five youth, three adult) who sustained a concussion reported symptom severity and number scores via the GSC during the early post-injury period (< 25 days) period. Four players (50%) reported symptom severity and six players (75%) reported symptom number changes greater than those suggested to be ‘broadly normal’ [35] following concussion. All players (100%) had returned to within normative normal ranges within the GRTP period [13].

## Discussion

This is the first study to use fNIRS to monitor early post-injury (< 25 days) and season long (8 months) changes in brain haemodynamic response utilising fNIRS measurements during activities inducing NVC and dCA as objective markers of brain health in adult and youth rugby players. Results showed no significant group differences between rugby and non-contact sport controls for changes in fNIRS measurements of NVC or dCA. There were, however, small but significant variation in adult and youth fNIRS responses during “Where’s Wally” and squat-stand tasks, indicating neuro-cognitive/physiological changes over time. Seven players (87.5%) who sustained a concussion showed altered fNIRS responses that were greater than the TE. Importantly, within the players who sustained a concussion, 75% of players’ NVC ∆O_2_Hb (Fig. 3a) and 87.5% of player’s ∆O_2_Hb SS (Fig. 4a fNIRS responses had not returned to normal levels within the GRTP period. Conversely, all players’ (100%) subjective symptom numbers and severity had returned to normal ranges within the same period (Table 4.).

### Playing Season

#### Neurovascular Coupling (NVC): “Where’s Wally”

No significant group differences were found for ∆O_2_Hb or ∆HHb between adult or youth rugby and control groups. However, significant differences during the season were seen with youth groups showing a decrease in ∆HHb from mid- to end-season. The “Where’s Wally” visual search task is designed to engage the visual and attention mechanisms in the brain [7, 18], resulting in increased neural activity. This requires more oxygen, leading to a process called neurovascular coupling [36]. In healthy individuals, complex visual search tasks induce an increase in O_2_Hb in the PFC [37]. The increase in ∆HHb seen within youth groups at mid-season indicates an altered haemodynamic response, with no change in task performance i.e., no change in number of correct identifications of “Where’s Wally” (Supplemental 1) to easily explain this alteration. Jain et al. [11] found that task familiarity significantly influenced PFC activation during dual-task assessments, with adolescents showing reduced O_2_Hb as they became more accustomed to repeated trials. This reduction suggesting a shift toward more efficient neural processing with experience. In the present study, the observed variability in ∆HHb among both youth groups over the season may reflect developmental differences in the brain’s ability to adapt to repeated cognitive demands, even in the absence of performance changes. While limited comparative studies exist, studies have reported significantly increased post-season ∆HHb and ∆total haemoglobin (∆tHb) in collegiate rugby players [38] and significantly increased ∆O_2_Hb from pre-to post-season in soccer players [39]. Additionally, average head impact load was associated with greater neural activation, suggesting that cumulative head impacts altered NVC responses [39]. The present study shows small but significant changes in NVC responses for both youth groups, indicating that the fNIRS changes are not due to playing rugby and accumulative impacts. As such our findings do not support previous literature [38, 39] that suggest changes in brain function in team sports outside of specific concussive events. Youth participants showed a more variable seasonal profile than adults, possibly because the PFC does not fully develop until the age of ∼ 25 years [30], with increases in PFC activity shown during a working memory task across 7 to 22 years [31]. This highlights the importance of developmental considerations when directly measuring concussive biomarkers.

#### Dynamic Cerebral Autoregulation (dCA): Squat-Stand

A squat-stand manoeuvre was used to acutely activate the dCA mechanism, which provides information on the brain’s ability to control and maintain cerebral blood flow (CBF) [40]. Healthy individuals are able to regulate CBF during orthostatic challenge [41] and no significant differences were seen in adult groups. Adult rugby players showed a trend towards a greater ∆O_2_Hb and ∆HHb during the squat-stand task compared to controls, possibly an effect of training [42]. Youth responses were again more variable, with pre-season ∆O_2_Hb and ∆HHb significantly lower than mid and end-season. Additionally, a significant interaction effect showed that youth control subjects produced higher values at mid-season compared to youth rugby players. This is not easily explained, although it must be noted that the ability to perform the squat-stand manoeuvre well was most challenging in this group. The variability in responses between adults and youths could reflect developmental differences in autoregulatory mechanisms [43, 44], indicating a need for age-specific considerations in assessing and understanding cerebrovascular responses to sport concussion.

Scarce comparative data are available. Studies have reported significant acute alterations in dCA in professional [45] and amateur boxers following sparring [46]. Additionally, significantly increased ∆O_2_Hb in the left PFC in contact sport athletes with a history of concussion [28], and reduced capacity to respond to dynamic changes in CBF over a season in professional rugby [47] and contact sport athletes [48] have been observed. Wallis et al. [46], using the same squat-stand manoeuvre as this study, measured middle cerebral artery blood velocity (MCAv) via transcranial Doppler ultrasound, and observed a significant reduction in phase (timing offset between mean arterial pressure and MCAv) post-boxing. The magnitude of the change was associated with the number of head impacts received and not seen following pad sparring without head impacts. Similarly, Wright et al. [48] applied the same squat-stand protocol and reported significant reductions in phase and increases in gain (response amplitude) over a season of contact sports participation. These changes were linked to cumulative sub-concussive exposure, suggesting that repetitive impacts can impair the cerebrovascular pressure-buffering system. The difference and stability of adult dCA responses in the present study may be the result of differing methods to assess CBF responses and/or related to the population. Collectively, our findings, alongside those discussed, underscore the utility of measuring mechanisms that assess the brain’s ability to control and regulate cerebral blood flow (CBF). The observed alterations in dCA across different sports contexts ranging from historical concussion to sub-concussive impacts highlight the need for ongoing cerebrovascular health monitoring in rugby and contact sport players.

### Concussion

#### NVC, dCA and GSC

Eight players experienced concussion and were monitored across the return-to-play period and at end-season. Seven out of eight players exhibited ∆O_2_Hb and ∆HHb responses during NVC and dCA inducing tasks outside normally expected values i.e., TE. Dysregulated NVC appears to manifest as either reduced ∆O_2_Hb or increased ∆O_2_Hb during the cognitive task. Reduced ∆O_2_Hb suggests that the brain’s ability to increase blood flow to active regions is compromised post-concussion, while increased ∆O_2_Hb may reflect the recruitment of additional neural resources or compensatory mechanisms. Both patterns are considered abnormal or dysregulated responses, indicating disruption of typical NVC. Importantly, these variations highlight the individual nature of NVC responses post-concussion, rather than a singular ‘negative’ or uniform pattern. Reduced cerebral activation using the same paradigm has been seen in retired rugby players with a history of concussion [49] and concussed youth sport players have shown differing brain responses, including hyperactivation during cognitive testing [50]. Additionally, concussed adolescents have also shown significantly reduced ∆ in the difference between O_2_Hb and HHb during a visual-cognitive task [51], which concurs with our findings.

Dysregulation was also seen during the squat-stand manoeuvre form the app six players (four youth, two adult) showing increased and one youth player reduced ∆O_2_Hb and ∆HHb. Retired contact sport athletes with a history of concussion have also shown significantly elevated ∆O_2_Hb in the left PFC [28] and dCA indices in concussed junior athletes, showed reduced phase (timing) [48]. The auto regulatory mechanism that manages CBF in response to changes in blood pressure appears to be compromised post-concussion, with concussed individuals displaying more pronounced responses to orthostatic challenge.

During the same time period, four players reported increased symptom severity and six players increased symptom number scores on the GSC, all greater than ‘broadly normal’ normative reported scores [35]. All symptoms had resolved within the GRTP period. Currently, resolution of symptoms forms the approach for clinical recovery within most amateur sports, despite evidence showing limited clinical application after seven-days post-injury [1].

Importantly, alterations in fNIRS responses during NVC (six out of eight ∆O_2_Hb; five out of eight ∆HHb) and dCA tasks (seven out of eight ∆O_2_Hb and ∆HHb) had not returned to within normal values within the GRTP period. Seven out of eight players returned to match play following clinical clearance, with four players’ NVC (∆O_2_Hb) and five players’ dCA (∆O_2_Hb) profiles outside of normal expected values at end-season. One player opted to take a prolonged recovery following concussive injury as they only had one game remaining for the season.

#### Implications

Our study offers critical insights into the neurocognitive/physiological impacts of a rugby season, with significant implications for player health monitoring, and concussion management. Firstly, we highlight the utility of fNIRS as an objective tool for monitoring cerebral oxygenation. The significant changes observed in NVC and dCA-inducing tasks, particularly among players who experienced concussions, underscore the potential of fNIRS measurements as biomarkers for brain health and concussion assessment. This supports a shift towards more objective, continuous monitoring of brain function in contact sports, rather than relying on subjective symptom reporting and clinical assessments [7, 48]. The persistence of altered fNIRS responses in concussed players beyond the GRTP period, despite the resolution of subjective symptoms, calls for a re-evaluation of current return-to-play guidelines. Incorporating objective measures such as fNIRS into return-to-play protocols could enhance the safety and health outcomes of rugby players. Additionally, age-specific considerations in concussion management, with tailored approaches to monitoring, and managing concussions is required.

### Strengths and Limitations

This study is among the first to provide both measurements of brain haemodynamic responses across a season and during the GRTP period post- concussion. The use of a portable, non-invasive technology has enabled field-based measurements which facilitates understanding of the effects of cumulative sub-concussive and concussive impacts. However, this study is not without limitations. Measurement, interpretation, and analysis of the fNIRS signal has important considerations; limited penetration depth to reach subcortical structures, limited coverage of the whole brain and confounders to the signal including movement artifact and physiological noise [52]. Stringent pre-processing steps, recommended by recent best practice fNIRS filtering approaches [53], were used to counter the influence of signal confounders. Despite widespread use within the sports science literature [33, 34], the use of TE to provide boundaries of accepted variation has not been employed within the context of fNIRS or concussion research previously. Future experimental designs should employ multiple individual pre-season measurements to establish individualised TE thresholds. Finally, the data analysis approaches presented here are limited to global measurements of the PFC. While this approach was appropriate for the aims of this study, more advanced analysis techniques, such as channel-by-channel or hemispheric-specific approaches, could provide greater insight into the spatial localisation of PFC activity. Such methods may be particularly useful for identifying specific brain regions affected by injury or for exploring potential lateralised effects associated with certain tasks. Future studies could incorporate these techniques to enhance the understanding of the neural mechanisms underlying concussion-related changes in PFC oxygenation.

## Conclusion

In conclusion, results indicate current return-to-play protocols are limited and underscore the need for an individualised approach to concussion management, utilising objective biomarker tools. Our findings demonstrate the effectiveness of fNIRS as a method for assessing brain haemodynamic response, and advocate for enhanced protocols that include baseline (pre-season) assessment to prioritise the long-term neurological health of players. This has broader applications for contact sports where players are at risk of concussive and sub-concussive impacts. Future research should continue to explore innovative methods for monitoring brain health in athletes, with a focus on developing safer sport practices and improving player welfare.

## Electronic Supplementary Material

Below is the link to the electronic supplementary material.

Supplementary Material 1

Supplementary Material 2

## Data Availability

The datasets used and/or analysed during the current study are available from the corresponding author on reasonable request.

## Abbreviations

CI: Confidence Intervals
CTE: Chronic Traumatic Encephalopathy
dCA: Dynamic Cerebral Autoregulation
fNIRS: Functional Near Infrared Spectroscopy
GHQ: General Health Questionnaire
GRTP: Graduated Return to Play Protocol
HHb: Deoxyhaemoglobin
MCAv: Middle Cerebral Artery blood velocity
mTBI: Mild Traumatic Brain Injury
NVC: Neurovascular Coupling
O_2_Hb: Oxyhaemoglobin
PFC: Pre-frontal Cortex
RFU: Rugby Football Union
SCAT5: Sport Concussion Assessment Tool Version 5
SD: Standard Deviation
SRC: Sport Related Concussion
SS: Squat Stand
TE: Typical Error
WW: “Where’s Wally”

## References

[1] Patricios JS, Schneider KJ, Dvorak J, Ahmed OH, Blauwet C, Cantu RC et al. Consensus statement on concussion in sport: the 6th International Conference on Concussion in Sport-Amsterdam, October 2022. Br J Sports Med. 2023;57(11):695–711. 10.1136/bjsports-2023-106898 37316210

[2] Batty GD, Frank P, Kujala UM, Sarna SJ, Valencia-Hernández CA, Kaprio J. Dementia and Alzheimer’s disease in former contact sports participants: population-based cohort study, systematic review, and meta-analysis. eClinicalMedicine. 2023;61:102056. 37425375 10.1016/j.eclinm.2023.102056 PMC10329127

[3] Bernick C, Hansen T, Ng W, Williams V, Goodman M, Nalepa B, et al. Concussion occurrence and recognition in professional boxing and MMA matches: toward a concussion protocol in combat sports. Phys Sportsmed. 2021;49(4):469–75. 33251911 10.1080/00913847.2020.1856631

[4] Mack CD, Solomon G, Covassin T, Theodore N, Cárdenas J, Sills A. Epidemiology of concussion in the National football league, 2015–2019. Sports Health. 2021;13(5):423–30. 33872087 10.1177/19417381211011446 PMC8404771

[5] King D, Hume PA, Brughelli M, Gissane C. Instrumented mouthguard acceleration analyses for head impacts in amateur rugby union players over a season of matches. Am J Sports Med. 2015;43(3):614–24. 25535096 10.1177/0363546514560876

[6] West SW, Cross M, Trewartha G, Taylor A, Brooks J, Kemp S, et al. Trends in match concussion incidence and return-to-play time in male professional rugby union: A 16-season prospective cohort study. Brain Inj. 2021;35(10):1235–44. 34495819 10.1080/02699052.2021.1972142

[7] Neary JP, Singh J, Bishop SA, Dech RT, Butz MJA, Len TK. An Evidence-Based objective study protocol for evaluating cardiovascular and cerebrovascular indices following concussion: the Neary protocol. Methods Protoc. 2019;2(1):23. 31164604 10.3390/mps2010023 PMC6481075

[8] NICE. Recommendations for research| Head injury: assessment and early management| Guidance| NICE [Internet]. NICE. 2023 [cited 2024 Apr 19]. Available from: https://www.nice.org.uk/guidance/ng232/chapter/Recommendations-for-research

[9] Murkin JM, Arango M. Near-infrared spectroscopy as an index of brain and tissue oxygenation. Br J Anaesth. 2009;103(Suppl 1):i3–13. 20007987 10.1093/bja/aep299

[10] Martini DN, Mancini M, Antonellis P, McDonnell P, Vitorio R, Stuart S, et al. Prefrontal cortex activity during gait in people with persistent symptoms after concussion. Neurorehabil Neural Repair. 2024;38(5):364–72. 38506532 10.1177/15459683241240423 PMC13089523

[11] Jain D, Graci V, Beam ME, Ayaz H, Prosser LA, Master CL, et al. Neurophysiological and gait outcomes during a dual-task gait assessment in concussed adolescents. Clin Biomech (Bristol). 2023;109:106090. 37696165 10.1016/j.clinbiomech.2023.106090 PMC10758982

[12] Hocke LM, Duszynski CC, Debert CT, Dleikan D, Dunn JF. Reduced functional connectivity in adults with persistent Post-Concussion symptoms: A functional Near-Infrared spectroscopy study. J Neurotrauma. 2018;35(11):1224–32. 29373947 10.1089/neu.2017.5365 PMC5962910

[13] England Rugby. Being RugbySafe (2022-23). 2022 [cited 2022 Dec 11]. Keep Your Boots On! Available from: https://keepyourbootson.co.uk/

[14] Howell DR, Kirkwood MW, Laker S, Wilson JC. Collision and contact sport participation and quality of life among adolescent athletes. J Athl Train. 2020;55(11):1174–80. 33112960 10.4085/1062-6050-0536.19 PMC7709212

[15] Echemendia RJ, Meeuwisse W, McCrory P, Davis GA, Putukian M, Leddy J, et al. The sport concussion assessment tool 5th edition (SCAT5): background and rationale. Br J Sports Med. 2017;51(11):848–50. 28446453 10.1136/bjsports-2017-097506

[16] Duncan A, Meek JH, Clemence M, Elwell CE, Fallon P, Tyszczuk L, et al. Measurement of cranial optical path length as a function of age using phase resolved near infrared spectroscopy. Pediatr Res. 1996;39(5):889–94. 8726247 10.1203/00006450-199605000-00025

[17] World Rugby. Concussion Guidance| World Rugby [Internet]. 2024 [cited 2024 Apr 19]. Available from: https://www.world.rugby/the-game/player-welfare/medical/concussion/concussion-guidelines

[18] Smirl JD, Wright AD, Bryk K, van Donkelaar P. *Where*’*s Waldo*? The utility of a complicated visual search paradigm for transcranial Doppler-based assessments of neurovascular coupling. J Neurosci Methods. 2016;270:92–101. 27291357 10.1016/j.jneumeth.2016.06.007

[19] Burma JS, Van Roessel RK, Oni IK, Dunn JF, Smirl JD. Neurovascular coupling on trial: how the number of trials completed impacts the accuracy and precision of temporally derived neurovascular coupling estimates. J Cereb Blood Flow Metab. 2022;42(8):1478–92. 35209741 10.1177/0271678X221084400 PMC9274868

[20] Claassen JAHR, Levine BD, Zhang R. Dynamic cerebral autoregulation during repeated squat-stand maneuvers. J Appl Physiol (1985). 2009;106(1):153–60. 18974368 10.1152/japplphysiol.90822.2008 PMC2636935

[21] Jasper HH. The Ten-Twenty electrode system of the international federation. Electroencephalogr Clin Neurophysiol. 1958;10:371–5. 10590970

[22] Abraham A, Pedregosa F, Eickenberg M, Gervais P, Mueller A, Kossaifi J et al. Machine learning for neuroimaging with scikit-learn. Front Neuroinform [Internet]. 2014 Feb 21 [cited 2024 Apr 19];8. Available from: https://www.frontiersin.org/articles/10.3389/fninf.2014.00014 10.3389/fninf.2014.00014 PMC3930868 24600388

[23] Pinti P, Scholkmann F, Hamilton A, Burgess P, Tachtsidis I. Current status and issues regarding Pre-processing of fNIRS neuroimaging data: an investigation of diverse signal filtering methods within a general linear model framework. Front Hum Neurosci. 2019;12:505. 30687038 10.3389/fnhum.2018.00505 PMC6336925

[24] Sappia MS, Hakimi N, Colier WNJM, Horschig JM. Signal quality index: an algorithm for quantitative assessment of functional near infrared spectroscopy signal quality. Biomed Opt Express BOE. 2020;11(11):6732–54. 33282521 10.1364/BOE.409317 PMC7687963

[25] Hu XS, Arredondo MM, Gomba M, Confer N, DaSilva AF, Johnson TD, et al. Comparison of motion correction techniques applied to functional near-infrared spectroscopy data from children. J Biomed Opt. 2015;20(12):126003. 26662300 10.1117/1.JBO.20.12.126003 PMC9900395

[26] Scholkmann F, Kleiser S, Metz AJ, Zimmermann R, Mata Pavia J, Wolf U, et al. A review on continuous wave functional near-infrared spectroscopy and imaging instrumentation and methodology. NeuroImage. 2014;85(Pt):6–27. 23684868 10.1016/j.neuroimage.2013.05.004

[27] Bishop SA, Neary JP. Assessing prefrontal cortex oxygenation after sport concussion with near-infrared spectroscopy. Clin Physiol Funct Imaging. 2018;38(4):573–85. 28626873 10.1111/cpf.12447

[28] Sirant LW, Singh J, Martin S, Gaul CA, Stuart-Hill L, Candow DG, et al. Long-term effects of multiple concussions on prefrontal cortex oxygenation during repeated squat-stands in retired contact sport athletes. Brain Inj. 2022;36(8):931–8. 35968581 10.1080/02699052.2022.2109737

[29] Sirant LW, Singh J, Martin S, Gaul CA, Stuart-Hill L, Candow DG, et al. Long-term effects of multiple concussions on prefrontal cortex oxygenation during neurovascular coupling activation in retired male contact sport athletes. Curr Res Physiol. 2022;5:421–8. 36466150 10.1016/j.crphys.2022.11.002 PMC9713254

[30] Johnson SB, Blum RW, Giedd JN. Adolescent maturity and the brain: the promise and pitfalls of neuroscience research in adolescent health policy. J Adolesc Health. 2009;45(3):216–21. 19699416 10.1016/j.jadohealth.2009.05.016 PMC2892678

[31] Kwon H, Reiss AL, Menon V. Neural basis of protracted developmental changes in visuo-spatial working memory. Proc Natl Acad Sci U S A. 2002;99(20):13336–41. 12244209 10.1073/pnas.162486399 PMC130634

[32] Roby PR, Mozel AE, Grady MF, Master CL, Arbogast KB. Neurovascular coupling in acutely concussed adolescent patients. J Neurotrauma. 2024;41(13–14):e1660–7. 38468544 10.1089/neu.2023.0192 PMC11564851

[33] Swinton PA, Hemingway BS, Saunders B, Gualano B, Dolan E. A statistical framework to interpret individual response to intervention: paving the way for personalized nutrition and exercise prescription. Front Nutr. 2018;5:41. 29892599 10.3389/fnut.2018.00041 PMC5985399

[34] Weatherwax RM, Harris NK, Kilding AE, Dalleck LC. Using a site-specific technical error to Establish training responsiveness: a preliminary explorative study. Open Access J Sports Med. 2018;9:47. 29563845 10.2147/OAJSM.S155440 PMC5848661

[35] Black AM, Miutz LN, Kv VW, Schneider KJ, Yeates KO, Emery CA. Baseline performance of high school rugby players on the sport concussion assessment tool 5. J Athl Train. 2020;55(2):116–23. 31917599 10.4085/1062-6050-123-19 PMC7017900

[36] Chen WL, Wagner J, Heugel N, Sugar J, Lee YW, Conant L, et al. Functional Near-Infrared spectroscopy and its clinical application in the field of neuroscience: advances and future directions. Front Neurosci. 2020;14:724. 32742257 10.3389/fnins.2020.00724 PMC7364176

[37] Chen LC, Sandmann P, Thorne JD, Herrmann CS, Debener S. Association of concurrent fNIRS and EEG signatures in response to auditory and visual stimuli. Brain Topogr. 2015;28(5):710–25. 25589030 10.1007/s10548-015-0424-8

[38] Clark A. Changes in cognitive function and cerebral oxygenation patterns in rugby and non-contact sportspersons over a 15-week season [Internet]. Stellenbosch: Stellenbosch University; 2018 [cited 2024 Apr 19]. Available from: http://hdl.handle.net/10019.1/103656

[39] Jain D, Huber CM, Patton DA, McDonald CC, Wang L, Ayaz H, et al. Use of functional near-infrared spectroscopy to quantify neurophysiological deficits after repetitive head impacts in adolescent athletes. Sports Biomech. 2023;0(0):1–15. 10.1080/14763141.2023.2229790 PMC10776807 37430440

[40] Golding EM, Robertson CS, Bryan RM. The consequences of traumatic brain injury on cerebral blood flow and autoregulation: a review. Clin Exp Hypertens. 1999;21(4):299–332. 10369378 10.3109/10641969909068668

[41] Kim JM, Choi JK, Choi M, Ji M, Hwang G, Ko SB, et al. Assessment of cerebral autoregulation using continuous-wave near-infrared spectroscopy during squat-stand maneuvers in subjects with symptoms of orthostatic intolerance. Sci Rep. 2018;8(1):13257. 30185974 10.1038/s41598-018-31685-y PMC6125591

[42] Roy MA, Labrecque L, Perry BG, Korad S, Smirl JD, Brassard P. Directional sensitivity of the cerebral pressure-flow relationship in young healthy individuals trained in endurance and resistance exercise. Exp Physiol. 2022;107(4):299–311. 35213765 10.1113/EP090159

[43] Armstead WM. Cerebral blood flow autoregulation and dysautoregulation. Anesthesiol Clin. 2016;34(3):465–77. 27521192 10.1016/j.anclin.2016.04.002 PMC4988341

[44] Vavilala MS, Newell DW, Junger E, Douville CM, Aaslid R, Rivara FP, et al. Dynamic cerebral autoregulation in healthy adolescents. Acta Anaesthesiol Scand. 2002;46(4):393–7. 11952439 10.1034/j.1399-6576.2002.460411.x

[45] Bailey DM, Jones DW, Sinnott A, Brugniaux JV, New KJ, Hodson D, et al. Impaired cerebral haemodynamic function associated with chronic traumatic brain injury in professional boxers. Clin Sci (Lond). 2013;124(3):177–89. 22913765 10.1042/CS20120259

[46] Wallis WEG, Al-Alem Q, Lorimer H, Smail OJ, Williams GKR, Bond B. The acute influence of amateur boxing on dynamic cerebral autoregulation and cerebrovascular reactivity to carbon dioxide. Eur J Appl Physiol. 2024;124(3):993–1003. 37768343 10.1007/s00421-023-05324-y PMC10879355

[47] Owens TS, Calverley TA, Stacey BS, Iannatelli A, Venables L, Rose G, et al. Contact events in rugby union and the link to reduced cognition: evidence for impaired redox-regulation of cerebrovascular function. Exp Physiol. 2021;106(9):1971–80. 34355451 10.1113/EP089330

[48] Wright AD, Smirl JD, Bryk K, Fraser S, Jakovac M, van Donkelaar P. Sport-Related concussion alters indices of dynamic cerebral autoregulation. Front Neurol. 2018;9:196. 29636724 10.3389/fneur.2018.00196 PMC5880892

[49] Sharma A, Hind K, Hume P, Singh J, Neary JP. Neurovascular coupling by functional near Infra-Red spectroscopy and Sport-Related concussion in retired rugby players: the UK rugby health project. Front Hum Neurosci. 2020;14:42. 32116616 10.3389/fnhum.2020.00042 PMC7033387

[50] Urban K, Schudlo L, Keightley M, Alain S, Reed N, Chau T. Altered brain activation in youth following concussion: using a Dual-task paradigm. Dev Neurorehabil. 2021;24(3):187–98. 33012188 10.1080/17518423.2020.1825539

[51] Master C, Storey E, Wang L, Grady M, McDonald C, Margulies S, et al. A functional near infrared spectroscopy investigation of the physiological underpinnings of visual cognitive workload after concussion. Orthop J Sports Med. 2022;10(5 suppl2):2325967121S00406.

[52] Tachtsidis I, Scholkmann F. False positives and false negatives in functional near-infrared spectroscopy: issues, challenges, and the way forward. Neurophotonics. 2016;3(3):031405. 27054143 10.1117/1.NPh.3.3.031405 PMC4791590

[53] Yücel MA, Lühmann Av, Scholkmann F, Gervain J, Dan I, Ayaz H, et al. Best practices for fNIRS publications. Neurophotonics. 2021;8(1):012101. 33442557 10.1117/1.NPh.8.1.012101 PMC7793571

