# Correction: Cerebral Haemodynamic Assessment Following Sport-related Concussion (Mild Traumatic Brain Injury) in Youth and Amateur Rugby Union Players

**DOI:** 10.1186/s40798-025-00866-1

**Published:** 2025-05-13

**Authors:** Ben Jones, Mohammadreza Jamalifard, Sally Waterworth, Mike Rogerson, Javier Andreu-Perez, Jay Perrett, Edward Hope, Jason Moran, Tom Adams, Jyotpal Singh, Patrick Neary, Chris E. Cooper

**Affiliations:** 1 School of Sport Rehabilitation and Exercise Sciences, University of Essex, Colchester, UK; 2 School of Computer Science and Electronic Engineering, University of Essex, Colchester, UK; 3 PhysiGo, Carlton Business Centre, Wiltshire, UK; 4 School of Sport and Exercise Sciences, Liverpool John Moores University, Liverpool, UK; 5 Faculty of Kinesiology & Health Studies, University of Regina, Regina, SK S4S 1A2 Canada

The authors wish to note an amendment to the following sentence in the original article:

‘Dysregulation was also seen during the squat-stand manoeuvre form the app six players (four youth, two adult) showing increased and one youth player reduced ΔO2Hb and ΔHHb.’

The statement should instead read as follows:

‘Dysregulation was also seen during the squat-stand manoeuvre with six players (four youth, two adult) showing increased and one youth player reduced ∆O2Hb and ∆HHb.’

