# Supplementary material for: Cerebral Haemodynamic Assessment Following Sport-related Concussion (Mild Traumatic Brain Injury) in Youth and Amateur Rugby Union Players
Source: Sports Med Open. 2025 May 2;11:47. doi: 10.1186/s40798-025-00849-2 (PMC12048381; doi:10.1186/s40798-025-00849-2)
Supplement: Supplementary file 1 — Supplementary Material 1 [file 40798_2025_849_MOESM1_ESM.docx]

Table 1. Group mean ± SD number of where’s Wally correct responses

| **Group** | **Pre-season** | **Mid-Season** | **End-Season** |
| --- | --- | --- | --- |
| Adult Rugby | 4.7 ± 3.4 | 5.0 ± 3.3 | 5.3 ± 3.0 |
| Adult Control | 4.9 ± 3.1 | 5.3 ± 3.5 | 4.0 ± 2.9 |
| Youth Rugby | 5.1 ± 3.5 | 5.4 ± 3.2 | 4.7 ± 2.9 |
| Youth Control | 4.5 ± 3.1 | 4.4 ± 3.3 | 4.0 ± 3.1 |

No significant differences were seen between response rates (p=>0.05)

Table 2. Concussed participants mean ± SD number of where’s Wally correct responses

| **Participant ID** | **Correct Response** |
| --- | --- |
| CR3 | 6.7 ± 1.4 |
| CR5 | 7.3 ± 1.6 |
| CR7 | 6.5 ± 1.9 |
| CR13 | 6.3 ± 3.0 |
| CR15 | 7.0 ± 1.1 |
| R13 | 6.2 ± 1.0 |
| R16 | 4.3 ± 2.6 |
| R18 | 6.2 ± 2.5 |

Note: Data shown is the average and SD taken over all visits in which the participants completed the Where’s Wally task
