# Supplementary material for: Cerebral Haemodynamic Assessment Following Sport-related Concussion (Mild Traumatic Brain Injury) in Youth and Amateur Rugby Union Players
Source: Sports Med Open. 2025 May 2;11:47. doi: 10.1186/s40798-025-00849-2 (PMC12048381; doi:10.1186/s40798-025-00849-2)
Supplement: Supplementary file 2 — Supplementary Material 2 [file 40798_2025_849_MOESM2_ESM.docx]

Table 1. shows all calculated technical error (TE) scores for “Where’s Wally” (WW) and squat stand (SS). Data are shown as mean with 90% confidence intervals (90%CI).

| **Group** | **O_2_Hb TE** | **HHb TE** |
| --- | --- | --- |
| Adult Rugby (WW) | 0.30 [0.24, 0.41] | 0.10 [0.08, 0.13]* |
| Youth Rugby (WW) | 0.42 [0.30, 0.95]* | 0.13 [0.10, 0.21] |
| Adult Control (WW) | 0.27 [0.21, 0.45] | 0.06 [0.04, 0.09] |
| Youth Control (WW) | 0.18 [0.14, 0.30] | 0.08 [ 0.06, 0.16] |
| Combined Adult WW | 0.29 [0.24, 0.37] | 0.08 [0.07,0.11] |
| Combined Youth (WW) | 0.32 [0.26, 0.44] | 0.11 [0.09, 0.14] |
| Adult Rugby (SS) | 1.66 [1.38, 2.23] | 0.32 [0.26, 0.44] |
| Youth Rugby (SS) | 1.38 [1.06, 2.02] | 0.20 [0.16, 0.30] |
| Adult Control (SS) | 1.34 [1.04, 1.97] | 0.19 [0.14, 0.29] |
| Youth Control (SS) | 1.37 [1.08, 1.92] | 0.19 [0.15, 0.27] |
| Combined Adult (SS) | 1.55 [1.31, 1.90] | 0.27 [0.23, 0.34] |
| Combined Youth (SS) | 1.41 [1.19, 1.80] | 0.23 [0.19, 0.29] |

*Denotes significant differences

Independent samples t-tests showed significant differences between control (adult and youth) and rugby participants TE scores for ∆HHb (adult) and ∆O_2_Hb (youth) “Where’s Wally” and no significant differences (p>0.05) for SS (∆O_2_Hb and ∆HHb) respectively.

*∆HHb Adult: t = 1.861 (20.062), p = 0.038, Cohens d = 0.662; *∆O_2_Hb Youth: t = 3.633 (14), p = 0.001, Cohens d = 1.816.
